# Isolation and Identification of Indigenous Wine Yeasts and Their Use in Alcoholic Fermentation

**DOI:** 10.17113/ftb.58.03.20.6677

**Published:** 2020-09

**Authors:** Polona Zabukovec, Neža Čadež, Franc Čuš

**Affiliations:** 1 Agricultural Institute of Slovenia, Department of Fruit Growing, Viticulture and Oenology, Hacquetova ulica 17, 1000 Ljubljana, Slovenia; 2 Department of Food Science and Technology, Chair of Biotechnology, Microbiology and Food Safety, Biotechnical Faculty, Jamnikarjeva ulica 101, 1000 Ljubljana, Slovenia

**Keywords:** spontaneous alcoholic fermentation, non-*Saccharomyces* yeasts, *Saccharomyces* yeasts, starter cultures, wine aroma compounds

## Abstract

**Research background:**

In our study, spontaneous alcoholic fermentations were carried out to isolate non-*Saccharomyces* and *Saccharomyces* yeasts from grape must from different vine-growing regions in Slovenia. Additionally, the diversity of native *Saccharomyces cerevisiae* strains was evaluated during the process.

**Experimental approach:**

During spontaneous alcoholic fermentations the yeast population of non-*Saccharomyces* and *Saccharomyces* yeasts was sampled. We used eleven microsatellite markers to determine the genetic diversity of *S. cerevisiae* strains. In addition, different ratios of the indigenous strains of *S. cerevisiae*, *Hanseniaspora uvarum* and *Starmerella bacillaris* were tested for their possible use in alcoholic fermentation with inoculated yeasts by monitoring its course and measuring the concentration of aroma compounds in wine.

**Results and conclusions:**

Sequencing of the internal transcribed spacer (ITS) regions of ribosomal DNA showed that of 64 isolates, 46 strains represent *S. cerevisiae* and 18 strains belong to non-*Saccharomyces* yeasts. The identified non-*Saccharomyces* yeast species were *H. uvarum*, *Pichia kudriavzevii*, *Saturnispora diversa* and *S. bacillaris*. The dendrogram grouped *S. cerevisiae* strains into 14 groups. The number of *S. cervisiae* strains isolated from the musts was 10 (Posavje), 11 (Podravje) and 25 (Primorska vine-growing region). On the other hand, the alcoholic fermentation with inoculated yeasts, in which the native *S. cerevisiae* strain predominated over *H. uvarum* and *S. bacillaris*, gave the most promising result due to the highest alcoholvolume fraction, the lowest acetic acid concentration and significantly higher concentrations of volatile thiols 3-mercaptohexyl acetate (3MHA) and 3-mercaptohexan-1-ol (3MH), 2-methylpropanol, 2-methylbutanol, 3-methylbutanol and 2-phenylethanol) in the produced wine.

**Novelty and scientific contribution:**

We confirmed the potential use of indigenous *S. cerevisiae* and non-*Saccharomyces* yeasts in alcoholic fermentation with inoculated yeasts, which allows the positive properties of the yeast strains to be expressed and good quality wines to be produced. Thus, the results are encouraging for winemakers to create different wine styles associated with a particular terroir using indigenous yeasts.

## INTRODUCTION

Spontaneous alcoholic fermentation is a process of many biochemical changes, due to external physical factors and the biological activities of fermenting microorganisms that include various species/strains of non-*Saccharomyces* and *Saccharomyces* yeasts (*1*, *2*). It usually begins with non-*Saccharomyces* yeasts, and the mainly present genera are *Hanseniaspora*, *Starmerella*, *Pichia*, *Debaryomyces* and *Metschnikowia*. They prevail on the surface of grape berries, and have a weak ability to ferment sugars in the must (*3*, *4*). When the fermentation begins, the exponential growth phase of the genera *Hanseniaspora*, *Candida* and *Pichia* yeasts is limited from two to three days, and after that, they reach a stationary phase. At later stages of the alcoholic fermentation, *S. cerevisiae* dominates the non-*Saccharomyces* strains, and completes the fermentation.

In spontaneous alcoholic fermentation, indigenous yeasts add the desired specific regional characteristics, but on the other hand, they might increase the risk of a stuck fermentation (*5*, *6*). The microorganisms present in the wine influence its chemical composition, and among them yeasts, especially *S. cerevisiae*, play the key role, leading the alcoholic fermentation. In stressful conditions of winemaking, *S. cerevisiae* show better adaptation to the winemaking conditions by growing faster and with high biomass productivity, which is correlated to higher viability in the late fermentation phases than with strains isolated from other environments (*1*). The main reasons for the dominance of *S. cerevisiae* yeast during alcoholic fermentation are the resistance to higher volume fractions of ethanol, and the ability to grow under anaerobic conditions (*7*, *8*). The *S. cerevisiae* strains from different genetical backgrounds differ in these characteristics and play an important role in determining the sensory quality of wine (*6*, *9*).

Indigenous *Saccharomyces* yeasts isolated from grapes can emphasise the specificity of the terroir, and can contribute to an increased market visibility of wine, due to their production of aromatic compounds which are formed during the fermentation, including higher alcohols, esters, terpenes and volatile thiols (*8*, *9*). With spontaneous alcoholic fermentation, we can obtain a greater quantity of compounds that significantly affect the sensory properties of the wine, which in general, have lower alcohol and/or residual sugar concentrations (*10*).

The quality of the wine produced by spontaneous alcoholic fermentation depends on the microbial population ecology of the grapes (*6*). Its characteristic is that indigenous yeast strains are better adapted to the chemical and microbiological properties of must in a given ecological environment (*11*, *12*). The grapevine cultivar, viticultural and oenological practices, macro- and microclimatic conditions and the geographic location of the vineyards mainly affect yeast biodiversity (*13*-*15*). In Slovenia, there are three vine-growing regions, Primorska, Podravje and Posavje with different pedoclimatic conditions influencing grapevine. Different vine-growing regions and grapevine cultivars may also delimit yeast populations and affect the genetic and phenotypic diversity of the yeasts (*14*, *16*, *17*).

Various studies have shown significant molecular polymorphisms of the indigenous *S. cerevisiae* strains from different vine-growing regions, and a strong correlation between their genomic and phenotypic properties (*13*, *18*-*20*). These yeasts might be better adapted to the fermentation of a particular grape and contribute to the typical oenological characteristics of a particular region (*2*). By using new tools for determining the wine yeast biodiversity, we can today better predict the characteristics of the wines with regard to terroir (*21*, *22*). Therefore, research on wine yeast biodiversity should be further implemented with new molecular and oenological approaches to enable winemakers to mimic spontaneous alcoholic fermentation, which would preserve the distinctive characteristics of their wines in correlation with the terroir properties.

Therefore, the objective of the study is to isolate indigenous non-*Saccharomyces* and *Saccharomyces* wine yeast strains from the must derived from three Slovenian vine-growing regions for their potential use as regional starter cultures and consequently to obtain wines with certain sensory characteristics that can be linked to the terroir.

## MATERIALS AND METHODS

### Grape sampling and spontaneous alcoholic fermentation

Undamaged and healthy grapes of different varieties, Malvazija (syn. Malvasia), Merlot, Refošk (syn. Refosco), Chardonnay, Šipon (syn. Furmint), Zweigelt, Modra frankinja (syn. Blaufränkisch) and Kerner were aseptically collected on different sampling dates in September 2016 (Table 1) in sterile plastic bags in three replicates (approx. 2 kg per replicate) from the vine-growing regions of Primorska (3 varieties×3=9 samples), Posavje (2 varieties×3=6 samples) and Podravje (4 varieties×3=12 samples), and processed separately in the laboratory.

**Table 1:** List of grape varieties by vine-growing region, location, sampling date and chemical parameters in must

Vine-growing region	Sampling location	Sampling date	Variety	*γ*(sugar)/(g/L)	*γ*(total acidity)/ (g/L)	pH
Primorska	a	8.9.2016	MAL	252.5±9.4	4.4±0.2	3.61±0.08
		8.9.2016	MER	237.0±8.8	4.2±0.3	3.48±0.09
	b	14.9.2016	R	272.2±31.6	7.6±0.6	3.18±0.07
						
Posavje	c	15.9.2016	K	240.7±32.3	5.6±0.3	3.21±0.04
		15.9.2016	MF	208.2±5.2	6.5±0.3	3.26±0.04
						
Podravje	d	20.9.2016	CH	214.5±1.3	5.6±0.6	3.33±0.04
	e	26.9.2016	Š	160.0±3.9	8.9±0.9	3.13±0.02
	f	26.9.2016	ZW	202.0±2.5	5.6±0.1	3.40±0.01
		26.9.2016	MF	191.1±12.9	8.2±0.7	3.13±0.09

Sampling locations: a=Debeli Rtič, b=Škofije, c=Pleterski hrib, d=Svetinje, e=Ivanjkovci, f=Mačkovci;

varieties: MAL=Malvasia, MER=Merlot, R=Refosco, K=Kerner, MF=Blaufränkisch,

CH=Chardonnay, Š=Furmint, ZW=Zweigelt, MF=Blaufränkisch

The must was treated with Redox Arom, a mixture of antioxidants: l-ascorbic acid 35%, K-metabisulphite 55% and purified gallotannins 10% (DAL CIN GILDO S.p.A., Concorezzo, Italy) to protect it from the action of oxygen in the must. The mixture was added into the bags in the amount of 0.2 g/kg of grapes. The grape juice was obtained after destemming, crushing and squeezing the grapes in a sterile inox container.

The must was poured into 1-litre sterile fermentors. Spontaneous alcoholic fermentations were performed at room temperature (21-23 °C), monitored by weighing the fermentors and calculating the amount of released CO_2_.

### Measurement of must parameters

Sugar concentration, total acidity and pH value were determined in the musts. The sugar concentration was measured by WineLab touch (CDR s.r.l., Ginestra Fiorentina, Florence, Italy). The total acidity and pH values were determined by the methods accredited in the laboratory (OIV-MA-AS313-01:R2015 (*23*) and OIV-MA-AS313-15:R2011 (*24*)).

### Yeast sampling, isolation and identification

During the spontaneous alcoholic fermentation, must aliquots were aseptically sampled and expected countable dilutions were plated on Wallerstein Laboratory (WL) nutrient agar (Merck, Darmstadt, Germany) in two replicates, in order to determine colony counts and to morphologically distinguish between non-*Saccharomyces* and *Saccharomyces* yeasts. The first sampling was carried out on the fifth day of spontaneous alcoholic fermentation, in order to obtain a first count of the non-*Saccharomyces* yeast population. Further samplings followed the dynamics of fermentations, and were taken in the middle and at the end of the fermentations.

After incubation at 26 °C for 2-3 days, the colonies were counted, and grouped depending on their morphology (OIV-MA-AS4-01:R2010) (*25*). Selected representatives of different morphological groups of yeasts were purified on yeast-malt (YM) agar plates (yeast extract 3.0 g/L (Biolife, Milano, Italy), malt extract 3.0 g/L (Biolife), peptone 5.0 g/L (Biolife), glucose 30.0 g/L (Merck), agar 20.0 g/L (Biolife)), with 0.01% chloramfenicol (Merck) added. The strains were cryopreserved in 10% glycerol (Merck) at −80 °C.

### Molecular identification of yeast species

The total DNA was isolated using the MasterPure™ Yeast DNA Purification Kit (Illumina, San Diego, CA, USA). The primers used for amplification of Iinternal transcribed spacer (ITS) regions and D1/D2 region of large subunit (LSU) rDNA were ITS1 (5` TCCGTAGGTGAACCTGCGG) and NL4 (5` GGTCCGTGTTTCAAGACGG) as described, respectively, by White *et al*. (*26*) and Kurtzman and Robnett (*27*). The final volume of the polymerase chain reaction (PCR) on the mixture was 50 µL containing 100 ng genomic DNA, 1×standard buffer Mg^2+^ free, 1.5 mM MgCl_2_, 2 mM of each dNTP, 50 pM of each of a pair of primers and 1 U Taq DNA polymerase (Promega, Madison, WI, USA).

For amplification of ITS rDNA, the PCR conditions were as follows: an initial denaturing step of 5 min at 94 °C was followed by 35 cycles of 40 s at 94 °C, 40 s at 56 °C and 30 s at 72 °C and terminated with a final extension step of 7 min at 72 °C and cooling down to 4 °C. The amplicon was sequenced by the commercial sequencing facilities (Macrogen Inc., Amsterdam, The Netherlands). The sequences were aligned and trimmed using BioNumerics v. 7.6 (*28*). For the molecular identification of yeast isolates, BLAST tools against GenBank recordings (*29*) of the rDNA sequences of the reference/type strains were used. All different sequences of the isolates were deposited in the GenBank and their accession numbers are listed in Table S1.

### Analysis of microsatellite repeats

To evaluate the genetic diversity of *S. cerevisiae* strains, their microsatellite regions were analysed according to Legras *et al*. (*30*). Two multiplex PCR reactions for amplification of loci C5, C3, C8, C11, SCYOR267c and YKL172w, ScAAT1, C4, SCAAT5, C6, YPL009c by using Qiagen Multiplex Master Mix (Qiagen, Hilden, Germany) were performed. The size of the fluorescently labelled PCR products was determined by capillary gel electrophoresis in a commercial laboratory (Macrogen, Seoul, South Korea). The number of repeats in the microsatellite loci was automatically determined by a multiple-locus variable-number tandem repeat analysis (MLVA) module of the BioNumerics v. 7.6 software (*28*). The results were then manually curated. A dendrogram of similarity was created using the Bay-Curtis algorithm and clustering unweighted pair group method with arithmetic mean analysis (UPGMA). All isolates were genotyped using eleven microsatellite loci.

### Measurement of chemical parameters of wine

After the completion of alcoholic fermentation, 2 mL/L of 5-6% sulphuric acid (Agrolit, Litija, Slovenia) were added to each fermentor, and later, the wine was transferred into bottles and placed in cold storage for clarification. The principle chemical parameters of wine (alcohol, glycerol, acetic acid, total and free SO_2_) were measured after one month of storage by WineLab Touch (CDR s.r.l.), where the measurements are based on enzymatic reactions for a single parameter according to the manufacturer´s instructions.

### Mixed alcoholic fermentation using indigenous strains

In the following experiment, we tested the fermentation capacity and efficiency of three previously isolated indigenous strains of *Saccharomyces cerevisiae* (RM1), *Hanseniaspora uvarum* (RM2) and *Starmerella bacillaris* (RM3). They showed a good potential to form volatile compounds (thiols, ethyl acetate, acetaldehyde and higher alcohols) during spontaneous alcoholic fermentation of Moscato bianco must, from which they were isolated and identified (*9*). The fermentation performance of these three indigenous strains was tested in Sauvignon must, in the following experiments: experiment A (33% *S. cerevisiae*, 33% *H. uvarum*, 33% *S. bacillaris*), experiment B (20% *S. cerevisiae*, 40% *H. uvarum*, 40% *S. bacillaris*) and experiment C (80% *S. cerevisiae*, 10% *H. uvarum*, 10% *S. bacillaris*). The fermentations were performed in 1000 mL glass fermentors in two replicates at (17.5±0.5) °C. The must had the following parameters: sugar concentration 207 g/L, total acidity 6.2 g/L, yeast assimilable nitrogen concentration 208 mg/L, and pH=3.21. The alcoholic fermentations were monitored by measuring mass loss. Must aliquots for plating were aseptically taken at the beginning, in the middle, and at the end of fermentation, and then plated on WL nutrient agar (Merck KGaA), in order to morphologically distinguish between inoculated non-*Saccharomyces* and *Saccharomyces* yeasts.

### Measurement of aromatic compounds in wine

At the end of the alcoholic fermentation, the chemical parameters and the content of the aromatic compounds were measured in the wines. The basic chemical parameters of wine were measured by WineLab Touch (CDR s.r.l.), where measurement is based on enzymatic reactions. The concentrations of volatile thiols (4-mercapto-4-methyl-pentan-2-one (4MMP), 3-mercaptohexan-1-ol (3MH) and 3-mercaptohexyl acetate (3MHA)) were measured two weeks after the completion of the fermentation on the GC-MS system. Hydroxy mercury benzoate (5 mL of a 2-mM solution) and butylated hydroxylanisole (0.5 mL of a 0.02-mM solution) were added to 50 mL of the wine sample. After mixing for 1 min, internal standards 4-methoxy-2-methyl-2-mercaptobutane (4M2M2MB), deuterated 3-mercaptohexan-1-ol (d3MH) and deuterated 3-mercaptohexyl acetate (d3MHA) were added, and the procedure continued according to the method described in Jenko *et al*. (*31*).

Ethyl acetate, acetaldehyde and higher alcohols were measured using the methods described by Bavčar *et al*. (*32*). The wine samples were diluted (1:4) with water (Milli-Q, Milipore, Billerica, MA, USA) to achieve a 1:3 ratio between the liquid and the headspace of a 20-mL solid space microextraction (SPME) vial. The samples were incubated at 40 °C for 1 h, and adsorbed to a polydimethylsiloxane/divinylbenzene (PDMS/DVB) fibre (Supelco, Bellefonte, PA, USA). The compounds were identified and quantified with a gas chromatograph (Agilent 7890A; Agilent Technologies, Palo Alto, CA, USA), equipped with an automatic multipurpose sampler MPS 2 (Gerstel, Mülheim and der Ruhr, Germany) and coupled with a mass spectrometer (Agilent 5975C; Agilent Technologies).

### Statistical analysis

The results were tested for normality distribution by Shapiro-Wilk test and statistically analysed using ANOVA in Statgraphics® Centurion XVI software (*33*).

## RESULTS AND DISCUSSION

### Chemical composition of the musts

During the harvest, grape samples of the representative grape varieties for each vine-growing region in Slovenia were collected and processed. As detailed in Table 1, the chemical parameters of the musts differed depending on grape variety and vine-growing region. The lowest concentration of total acids in musts from white varieties and the highest concentrations of sugars were found in musts from the Primorska region, which has a Mediterranean climate. However, generally the highest total acid concentrations and the lowest densities were characteristic of the Podravje region musts, which have a cooler climate. If we take a closer look at the must parameters, they are mainly linked to the varietal characteristics, *e.g*. Refosco in Primorska, with a higher value for total acidity, and Kerner, Chardonnay and Zweigelt, with lower values in regions Posavje and Podravje.

### Fermentation kinetics of the musts

**Fig. 1:**
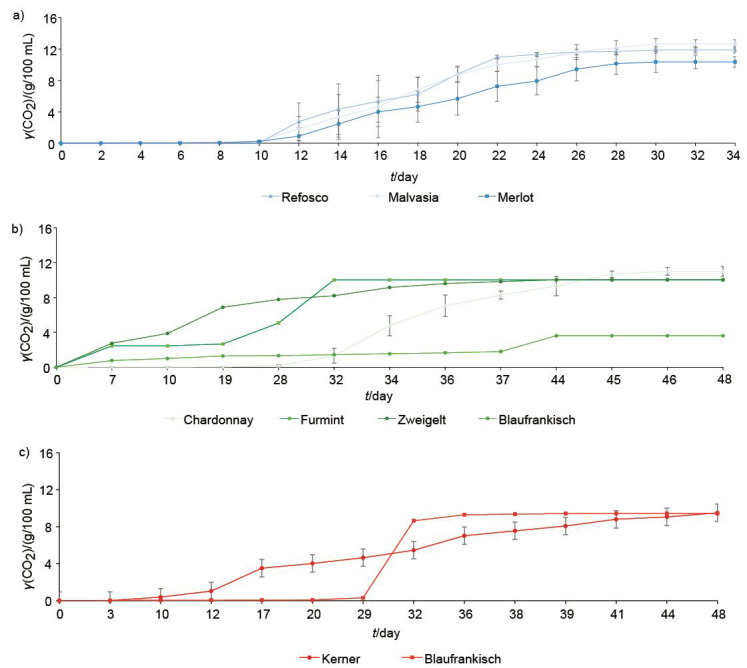
Mass concentration of exhausted CO_2_ in spontaneous alcoholic fermentation of: a) Refosco, Malvasia and Merlot (Primorska vine-growing region) (average values±S.D. are shown) b) Kerner (average values±S.D. are shown) and Blaufränkisch (one fermentation completed) (Posavje vine-growing region), c) Chardonnay (three fermentations completed), Zweigelt (two fermentations completed), Furmint (one fermentation completed), and Blaufränkisch (none fermentation completed) (Podravje vine-growing region) (average values±S.D. are shown for Chardonnay and Zweigelt)

Using the collected grape samples, 27 spontaneous alcoholic fermentations were conducted. The amount of exhausted CO_2_ was more intense in the fermentors with varieties from the Primorska vine-growing region, on average 12.49 g per 100 mL of must (Fig. 1a), which is also a consequence of higher initial sugar concentration in the must (Table 1). All spontaneously fermented wine samples from the Primorska vine-growing region had a content of reducing sugars below 42 g/L when the fermentation stopped. In the fermentors with the must from the Posavje vine-growing region (Fig. 1b), the average final mass of exhausted CO_2_ was 9.6 g and in the fermentors from the Podravje vine-growing region 8.7 g per 100 mL of must (Fig. 1c). The eight samples (two fermentors containing Blaufränkisch (MF) variety from the Posavje vine-growing region, and three fermentors with Blaufränkisch (MF) variety, two fermentors with Furmint (Š) variety and one fermentor with Zweigelt (ZW) variety from the Podravje vine-growing region) did not reach the stationary phase after 45 days of spontaneous alcoholic fermentation, because the fermentation got stuck.

The nutritional status of must is an important parameter that influences the alcoholic fermentation (*10*, *21*). In our case, we noticed the poor kinetics of alcoholic fermentations and yeast efficiency, probably due to the poor nutritional status of the must together with a specific yeast population that developed in each fermentor and led to the stuck fermentation.

### Abundance of yeasts and their identification

We isolated altogether 64 isolates for further identification: 46 *Saccharomyces* and 18 non-*Saccharomyces* yeast (Table S1) and determined their macro- and micromorphology. The count of yeasts was determined at different sampling points; however, none of the *Saccharomyces* and non-*Saccharomyces* yeasts were isolated from the third sampling (Table 2).

**Table 2:** Average yeast counts (*N*(log CFU/mL) in all spontaneous alcoholic fermentations from each region and the number of yeast isolates obtained at the beginning (I.), in the middle (II.), and at the end (III.) of all fermentations per region

Region/ Sampling	Primorska		Posavje				Podravje		
*N*(log CFU/mL)	*N*(*S.cerevisiae*)/ *N*(Non- *Saccharomyces*sp.)	*N*(log CFU/mL)	*N*(*S.cerevisiae*)/ *N*(Non-*Saccharomyces* sp.)		*N*(log CFU/mL)			*N*(*S. cerevisiae*/ *N*(Non-*Saccharomyces* sp.)
I.	3.6	0/2	3.3	0/6	3.8			0/10	
II.	6.5	25/0	5.7	10/0	6.4			11/0	
III.	6.1	0	0	0	5.9			0	

The percentage of similarity between the ITS and D1/D2 sequences of the species type strains and our isolates (representatives of each group) with the GenBank accession numbers is shown in Table S1. The identified non-*Saccharomyces* yeast species were *Hanseniaspora uvarum* (five strains), *Pichia kudriavzevii* (three strains), *Saturnispora diversa* (two strains), and *Starmerella bacillaris* (syn. *Candida zemplinina*) (eight strains). *H. uvarum* is one of the prevailing apiculate yeast species in wine grapes from warm vine-growing regions, and was isolated only from Merlot must at the beginning of alcoholic fermentation. Yanagida *et al*. (*34*) also found the species to be a typical constituent of the yeast microbiota under mild climatic conditions.

Non-*Saccharomyces* strains of oenological interest were isolated from the must of Furmint variety. Although the fermentation was stuck in two fermentors, we obtained three representatives of *H. uvarum* and three of *P. kudriavzevii*. Nemcová *et al*. (*35*) reported that in their research, *P. kudriavzewii* isolated from Blaufränkisch variety, was more associated with damaged than with intact grapes. The third most common non-*Saccharomyces* yeast was *S. bacillaris* and was isolated as a representative from Kerner must, three from Blaufränkisch must from Posavje and two from Podravje. We should stress that our intention was to get a diverse population of *S. cerevisiae* strains, and not to get an excess population of non-*Saccharomyces* isolates.

### Chemical composition of wines

When the concentration of reducing sugars dropped below 45 g/L, we stopped spontaneous alcoholic fermentation by adding sulphur to the wine (varieties from Primorska on the 34th day, varieties from Posavje on the 48th day and varieties from Podravje on the 46th day). Later on, some chemical components in the wines were measured. Table 3 shows the differences among the wines of different grape varieties, as a result of *Saccharomyces* and non-*Saccharomyces* yeasts present in the fermenting must. Interestingly, for all wine samples, the volatile acid concentration was below 1 g/L.

**Table 3:** Chemical composition of the wines obtained by spontaneous alcoholic fermentation

Vine region	Grape variety	*N*(isolated yeasts)		Chemical parameter							
*S.* *cerevisiae*	Non-*Saccharomyces* sp.	*φ*(alcohol)/ % vol.	*γ*(glycerol)/ (g/L)	*γ*(acetic acid)/(g/L)	*γ*(malic acid)/(g/L)	*γ*(free SO_2_)/(mg/L)	*γ*(total SO_2_)/(mg/L)	*γ*(reducing sugars)/(g/L)
Primorska	Malvasia*	7	0	14.7±0.7	8.8±0.8	0.7±0.1	1.4±0.5	4.7±1.2	76.0±16.4	39.7±4.0
	Merlot*	8	2	13.5±0.5	8.7±0.6	0.6±0.1	1.2±0.1	9.7±7.8	81.0±10.2	41.3±7.6
	Refosco*	10	0	13.9±0.1	5.4±4.7	0.4±0.1	3.4±0.5	9.3±1.2	87.0±7.2	7.0±6.2
Posavje	Kerner*	8	1	12.5±0.4	8.0±2.3	0.3±0.0	2.8±0.0	19.5±6.4	104.0±1.4	31.5±0.7
	Blaufränkisch***	2	5	11.8	7.9	0.2	2.5	13.0	102.0	29.0
Podravje	Chardonnay*	4	0	13.0±0.3	8.1±0.8	0.3±0.1	3.7±0.6	12.3±7.6	73.0±14.0	32.0±0.0
	Zweigelt**	7	0	12.0±0.3	10.0±2.3	0.2±0.0	3.0±0.1	18.0±2.8	135.5±6.4	34.0±4.2
	Furmint***	0	6	9.6	4.0	0.2	4.5	21.0	85.0	26.0

*Completion of three spontaneous alcoholic fermentations (average value±S.D.)

**Completion of two spontaneous alcoholic fermentations (average value±S.D.)

***Completion of one spontaneous alcoholic fermentation (only data for one fermentor are shown)

The variety where all three alcoholic fermentations were stuck is not shown (Blaufränkisch from Podravje, where additional 4 non-*Saccharomyces* yeasts were isolated)

### Genetic diversity of S. cerevisiae populations

**Fig. 2:**
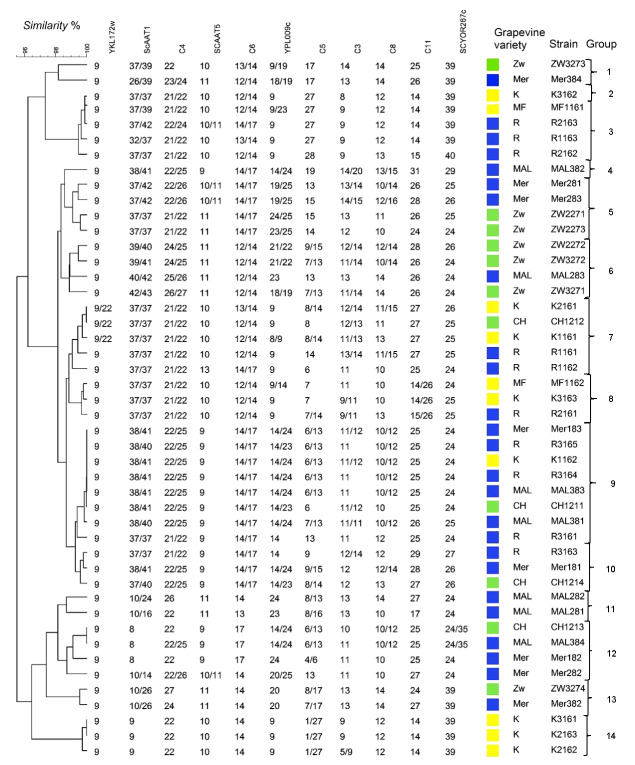
Dendrogram of the similarity of 46 *S.cerevisiae* strains isolated from different vine-growing regions: Primorska (blue square), Podravje (green square) and Posavje (yellow square) and varieties (MAL=Malvasia, MER=Merlot, R=Refosco, K=Kerner, MF=Blaufränkisch, CH=Chardonnay, ZW=Zweigelt), further analysed with 11 microsatellite loci (YKL172w, ScAAT1, C4, SCAAT5, C6, YPL009c, C5, C3, C8, C11 and SCYOR267c). The numbers below each microsatellite locus represent strain ploidy; homozygous strain has a single allele at a single microsatellite locus, and two different alleles present a heterozygous diploid

In order to investigate the genetic diversity of *S. cerevisiae*, polymorphisms in eleven microsatellite regions of 46 strains were determined, and are shown in the dendrogram of similarity (Fig. 2). The dendrogram clustered *S. cerevisiae* strains into 14 groups at a similarity level of 99%. As reported by Schuller *et al*. (*12*), many *S. cerevisiae* strains were homozygous, although in our case, many were heterozygous, indicating outcrossing between different populations.

The numbers of *Saccharomyces cerevisiae* strains isolated from the musts were 10 (Posavje), 11 (Podravje) and 25 (Primorska). We did not isolate *S. cerevisiae* strains from the spontaneous alcoholic fermentations of Furmint and Blaufränkisch must.

Genetically identical strains in all loci, *S. cerevisiae* R3164 and *S. cerevisiae* MAL383 (group 9), were isolated from two different vineyards in the Primorska vine-growing region, and were heterozygous in loci ScAAT1, C4, C6, YPL009c, C5 and C8. Interestingly, the strains *S. cerevisiae* Mer183 and *S. cerevisiae* K1162, belonging to group 9, were isolated from different vine-growing regions, and were identical in all loci. Group 9 comprised eight strains of *S. cerevisiae*, of which six strains were isolated from the Primorska vine-growing region. Genetically the most closely related strains from one variety in this group were isolated from the Refosco must. Otherwise, the genetically diverse group of strains (group 7), isolated from the varieties of the Primorska, Posavje and Podravje vine-growing regions, were identical in two motifs (37/37 and 21/22) of microsatellite loci ScAAT1 and C4. Genetically similar strains of group 14, *S. cerevisiae* K3161, *S. cerevisiae* K2163 and *S. cerevisiae* K2162, were isolated from the Posavje vine-growing region, and coincided in all microsatellite loci (K3161 and K2163), except C3 (K2162). *S. cerevisiae* MAL384 and *S. cerevisiae* CH1213 (group 12) were isolated from different vine-growing regions, and differed in C4 and C3 loci only.

Strains from the Primorska vine-growing region had characteristic microsatellite loci YKL172w and C4 with seven repeats of the motif (7 out of 13 strains). Strains from the Podravje vine-growing region also had the characteristic microsatellite loci YKL172w and ScAAT5, with eight repeats of the motif (8 out of 12 strains).

In Slovenian yeast populations, from two to 22 different alleles were detected in a single microsatellite locus. The largest number of alleles, and thus the highest diversity of *S. cerevisiae*, were shown by microsatellite loci C5, YPL009c, C3 and ScAAT1. As reported by other authors (*20*, *36*), microsatellite loci C3, C5 and ScAAT1 are most commonly used to describe the genotype of *S. cerevisiae* present in complex samples such as must.

### Chemical composition of the wines produced by indigenous yeast strains

**Fig. 3:**
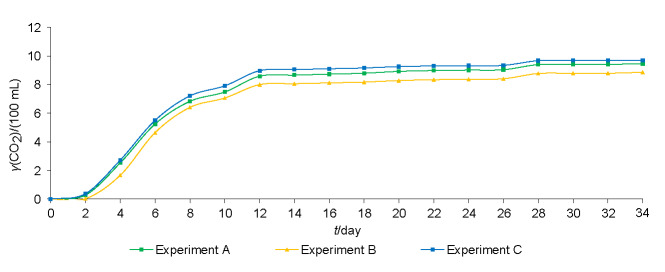
Mass concentration of exhausted CO_2_ during alcoholic fermentation inoculated with indigenous *Saccharomyces cerevisiae*, *Hanseniaspora uvarum* and *Starmerella bacillaris* strains at different initial ratios (in experiment A: 33:33:33, experiment B: 20:40:40 and experiment C: 80:10:10) in 1000-mL flasks (two replicates; average values±S.D. are shown)

To observe the ability and efficiency of isolated indigenous yeasts to conduct wine fermentations as starter cultures, we continued our study with three previously isolated yeasts of *Saccharomyces* and non-*Saccharomyces* yeasts. Fig. 3 shows the mass fraction of exhausted CO_2_ during alcoholic fermentation inoculated with different initial ratios of indigenous yeasts. As expected, the weakest fermentation kinetics in experiment B was with the lowest ratio of *S. cerevisiae* and *vice versa*, the most intensive in experiment C with the highest ratio of *S. cerevisiae*. Slightly less intensive was the fermentation kinetics in experiment A with an equal ratio of all three yeast species.

Alcoholic fermentations with different combinations of indigenous yeast strains were stopped after 35 days, when the concentration of reducing sugars was (10.8±0.0) g/L in experiments A and B and (10.7±0.1) g/L in experiment C (Table 4). The alcohol volume fraction was significantly lower in the fermentors with the lowest initial ratio of *S. cerevisiae* (experiment B) ((11.4±0.0) %). The differences in glycerol concentration were not significant, while the volatile acidity was significantly higher in experiment B ((0.86±0.0) g/L) and significantly lower in experiment C ((0.57±0.0) g/L) than in experiment A ((0.65±0.0) g/L). The significantly higher concentrations of free SO_2_ and total SO_2_ were in experiment A ((9.0±0.4) mg/mL and (61.0±1.2) mg/mL), where the ratio of yeasts was equal.

**Table 4:** Chemical composition of wines obtained in different experiments in 1000-mL fermentors

Chemical parameter	Experiment A	Experiment B	Experiment C	Sensory perception threshold
*φ*(alcohol)/%	(12.1±0.1)^b^	(11.4±0.0)^a^	(12.4±0.1)^b^	/
*γ*(glycerol)/(g/L)	(7.8±0.2)^a^	(7.6±0.1)^a^	(7.6±0.2)^a^	/
*γ*(acetic acid)/(g/L)	(0.65±0.00)^b^	(0.86±0.0)^c^	0(.57±0.0)^a^	/
*γ*(free SO_2_)/(mg/L)	(9.0±0.4)^b^	(7.0±0.8)^a^	(7.0±0.4)^a^	/
*γ*(total SO_2_)/(mg/L) *γ*(reducingsugars)/(g/L)	(61.0±1.2)^a^ (10.8±0.0)^a^	(57.0±0.8)^a^ (10.8±0.0)^a^	(60.0±1.2)^a^ (10.7±0.0)^a^	/
*γ*(volatile thiols)/(ng/L)				
3MHA	(234.0±77.8)^ab^	(169.0±35.5^)a^	(254.0±2.3)^b^	**4**
3MH	(2005±14)^b^	(1344±124)^a^	(2310±64)^b^	**60**
4MMP	(11.0±1.8)^a^	(31.0±19.4)^a^	(26.0±16.1)^a^	**0.8**
*γ*(acetaldehyde, ethylacetate and higher alcohols)/(mg/L)				
Acetaldehyde	(44.0±4.5)^a^	(40.0±1.0)^a^	(35.0±1.5)^a^	100-125
Ethylacetate	(48.0±2.5)^a^	(48.0±2.0)^a^	(48.0±2.5)^a^	**15**
1-propanol	(9.0±0.0)^a^	(9.0±1.0)^a^	(10.0±0.0^)a^	40
2-methylpropanol	(20.0±0.5)^b^	(16.0±0.0)^a^	(21.0±0.0)^b^	40
1-butanol	(3.0±0.0)^a^	(3.0±0.0)^a^	(3.0±0.0)^a^	30
2-methylbutanol	(27.0±1.0)^b^	(22.0±0.5)^a^	(31.0±0.0)^b^	**15**
3-methylbutanol	(94.0±4.5)^b^	(79.0±0.0)^a^	(105.0±0.0)^b^	**30**
2-phenylethanol	(69.0±1.0)^a^	(69.0±0.5)^a^	(82.0±3.0)^b^	**10**

Alcoholic fermentations inoculated with diffent ratios of indigenous *Saccharomyces cerevisiae*, *Hanseniaspora uvarum* and *Starmerella bacillaris* strains: experiment A=33:33:33, experiment B=20:40:40 and experiment C=80:10:10.

The letters in superscript in columns indicate statistically significant differences among experiments at a confidence level of 95%.

The values in bold indicate the compounds that exceed sensory perception threshold.

The concentrations of volatile thiols (3MHA, 3MH and 4MMP) were determined in the wine samples 14 days after the alcoholic fermentation was completed (Table 4). The concentrations of all three volatile thiols exceeded the limits of sensory perception in wine. The significantly lowest concentration of 3MHA was measured in experiment B (169 ng/L). The combination of yeasts in experiment B released the lowest concentrations of 3MH (1344 ng/L), compared to the other two combinations (2005 and 2310 ng/L, respectively). Yeasts in experiment C released significantly higher concentrations of 3MHA (254 ng/L), with *S. cerevisiae* being predominant; therefore, individual yeast strains have different genetic and psychological abilities to release volatile thiols from their precursors and to convert 3MH to 3MHA (*34*). For the concentration of 4MMP, no significant difference was confirmed. However, in experiment B the highest concentration of 4MMP was measured (31 ng/L), and in experiment A the lowest (11 ng/L). Numerous studies have shown that the amount of released 4MMP in wine depends on the strain of wine yeasts used for alcoholic fermentation (*37*, *38*).

The concentrations of ethyl acetate, acetaldehyde and higher alcohols in wines were measured 45 days after the completion of alcoholic fermentation (Table 4). At concentrations below 300 mg/L (*8*), higher alcohols gave the wines the desired complexity and improved their sensory quality, as it was noticed in all three experiments. The concentrations of ethyl acetate, 2-methylbutanol, 3-methylbutanol and 2-phenyl ethanol exceeded the sensory perception threshold in wine. Compounds 2-butanol and 2-propenyl alcohol in wines were below the limit of detection. Regardless of the composition of the yeast strains, the same amount of ethyl acetate was produced, although many studies have shown that more ethyl acetate is released in mixed alcoholic fermentations in which the ratio of non-*Saccharomyces* yeasts was higher (*39*). Various yeast strains affected the profile and concentration of the higher alcohols in the wine. The significantly higher concentrations of 2-methylpropanol, 2-methylbutanol, 3-methylbutanol and 2-phenylethanol were measured in experiment C, with the highest ratio of *S. cervisiae*. Moreira *et al.* (*40*) reported higher concentrations of 1-propanol, which is known for its harsh heavy odour, in mixed alcoholic fermentations with non-*Saccharomyces* and *Saccharomyces* yeasts. However, we measured concentrations of 1-propanol far below the sensory perception threshold.

## CONCLUSIONS

In this study, we observed the fermentative yeast microbiota during the spontaneous alcoholic fermentation of must of different grape varieties from three vine-growing regions in Slovenia. Our main objective was to obtain oenologically interesting strains for further use in inoculated alcoholic fermentations. The spontaneous alcoholic fermentations had different dynamics, and when stopped, different concentrations of unfermented sugars in the must were measured. Some spontaneous fermentations also got stuck. The result of such processes depends on the composition and initial count of the fermentative yeast species and strains and the must composition, especially the sugar content, yeast assimilable nitrogen and vitamin concentrations.

Furthermore, we evaluated eleven microsatellite markers to determine genetic diversity of 46 *S. cerevisiae* strains. From the obtained results it would be difficult to confirm a link between the location/region and the isolated *Saccharomyces cerevisiae* strains. Representatives of the same dendrogram groups or their close neighbouring groups occur in almost all vineyard areas. We have checked the databases and these are not known strains from yeast starter cultures.

Different initial ratios of indigenous *Saccharomyces* and non-*Saccharomyces* yeasts inoculated in the must influenced the fermentation kinetics and the concentration of volatile and non-volatile compounds in the wine. In our study, we confirmed the best results in a combination dominated by *S. cerevisiae* (80%) and *Hanseniaspora uvarum* and *Stamerella bacillaris* with 10% each, as this combination led to an appropriate volume fraction of alcohol, a lower concentration of acetic acid and the significantly highest concentrations of volatile thiols and higher alcohols. The combination with only 20% *S. cerevisiae* gave the lowest volume fraction of ethanol, which could be interesting for the production of low-alcohol wines. However, this combination produced the highest concentration of acetic acid and significantly lower concentrations of 3MHA, 3MH, 2-methylpropanol, 2-methylbutanol, 3-methylbutanol and 2-phenylethanol.

We confirmed the potential use of indigenous *S. cerevisiae* and non-*Saccharomyces* yeasts in inoculated alcoholic fermentations, which allows the positive properties of the yeast strains to be expressed and good quality wines to be produced. Thus, the results are encouraging for winemakers to create different wine styles associated with a particular terroir using indigenous yeasts.

## Figures and Tables

**Table S1:** Morphological characteristics of the yeast strains isolated on WL agar and their designation, sequence similarity with species type strain and GenBank (*29*) accesion numbers of ITS and D1/D2 sequences

Species	No.	Yeast strain	Colony colour	Colony morphology	*N*(compared nucleotide)	Sequence similarity/%	GenBank acc. no.
*S. cerevisiae*	1.	R2161	Cream to green	Grown in centre, smooth, opaque, butyrous		100	
	2.	K2161				100	
	3.	Mer382				100	
	4.	K3161			1312	99.3	MT734685
	5.	K3162				100	
	6.	R3165				100	
	7.	CH1213				100	
	8.	R3161				100	
	9.	R3163				100	
	10.	MAL382			1310	100	MT734684
	11.	ZW2271				100	
	12.	Mer384				100	
	13.	Mer182				100	
	14.	K1161			1313	99.8	MT734686
	15.	R1161				100	
	16.	CH1212				100	
	17.	Mer281				100	
	18.	R1163				100	
	19.	MAL282				100	
	20.	CH1211				100	
	21.	K3163			1312	99.8	MT734687
	22.	Mer282				100	
	23.	MAL381				100	
	24.	R2162				100	
	25.	ZW2273				100	
	26.	Mer283				100	
	27.	ZW2272				100	
	28.	R1162				100	
	29.	MAL384				100	
	30.	K2163				100	
	31.	R3164				100	
	32.	Mer181				100	
	33.	K1162				100	
	34.	MAL281				100	
	35.	MF1161				100	
	36.	ZW3271				100	
	37.	CH1214				100	
	38.	MAL283				100	
	39.	MAL383				100	
	40.	ZW3272				100	
	41.	MF1162				100	
	42.	Mer183				100	
	43.	R2163				100	
	44.	ZW3273				100	
	45.	ZW3274				100	
	46.	K2162				100	
*Hanseniaspora uvarum*	47.	Mer381	Intense green	Flat surface, smooth, opaque, butyrous		100	
	48.	Š3272				100	
	49.	Mer383			1251	100	MT734682
	50.	Š3271				100	
	51.	Š3273				100	
*Pichia kudriavzevii*	52.	Š2271	White to cream	Low convex with a flattened centre, lobed, fringed margin, butyrous	998	100	MT734683
	53.	Š2273				100	
	54.	Š2272				100	
*Saturnispora diversa*	55.	MF1163	White to cream	Smooth, butyrous, with entire margin	550	100	MT730589*
	56.	M1282				100	
*Starmerella bacillaris*	57.	M2281	Light to intense green	Low colonies with smooth to finely lobed margins, butyrous		100	
	58.	MF3163				100	
	59.	K2164				100	
	60.	MF3162			890	100	MT734681
	61.	M1281				100	
	62.	MF3161				100	
	63.	M2282				100	
	64.	MF3164				100	

*only D1/D2 region
